# Distorted assessment of left atrial size by echocardiography in patients with increased aortic root diameter

**DOI:** 10.1186/s43044-021-00177-2

**Published:** 2021-06-26

**Authors:** Abdullah Kaplan, Raffaele Altara, Marco Manca, Hacı Murat Gunes, Alessandro Cataliotti, George W. Booz, Fouad A. Zouein

**Affiliations:** 1 Department of Pharmacology and Toxicology, American University of Beirut Faculty of Medicine, Riad El-Solh, Beirut, 1107 2020 Lebanon; 2 Department of Cardiology, Medipol University, Sefakoy Hospital, Tevfik Bey, Maslak Cesme Cd., No:30, 34295 Kucukcekmece, Istanbul, Turkey; 3 Institute for Experimental Medical Research, Oslo University Hospital and University of Oslo, Oslo, Norway; 4 KG Jebsen Center for Cardiac Research, University of Oslo, Oslo, Norway; 5 Department of Pathology, School of Medicine, University of Mississippi Medical Center, Jackson, MS USA; 6 DG-DI, Medical Applications, CERN, 1211, 23 Geneva, Switzerland; 7 Department of Pharmacology and Toxicology, School of Medicine, University of Mississippi Medical Center, Jackson, MS USA

**Keywords:** Echocardiography, Aortic root dilation, Left atrial structure, Linear measurement of left atrium, Left atrial volume

## Abstract

**Background:**

Left atrial (LA) size is frequently assessed by posterior-anterior linear measurement of LA (LAD P-A) in the parasternal long axis to expedite examination. Aging, changes in body surface area, and several cardiovascular pathologies can affect aortic root (AoR) size, thereby affecting LA anatomical shape. We hypothesized that AoR dilatation influences LAD P-A and consequently correct assessment of LA size.

**Results:**

We tested our hypothesis in a study of 70 patients with AoR diameter ranging from 2.7 to 4.8 cm. LA size assessed in parasternal long axis view as LAD P-A was compared to that with LA width and length acquired in the apical two and four chamber view. Simpson’s method of discs was used as standard measurement to assess LA volume.

We observed that LAD P-A in the parasternal long axis decreases when AoR diameter increases. Thus, the increase in LA size assessed in parasternal long axis did not correlate with the increase of LA volume. Further analysis revealed that a significant positive correlation was observed when LAV was plotted as a function of LAD P-A only for those with a normal size AoR. In contrast, LA volume increase correlated with LA diameters assessed in the apical two and four chamber view regardless of AoR size.

**Conclusions:**

Our study documents that increases in AoR impact on the linear measurement of LA, resulting in an underestimated LAD P-A. LA size ought to be calculated from the apical two and four chambers view parameters, especially in patients with AoR dilatation.

## Background

Left atrium has an integral role in cardiac performance [1, 2]. Regardless of the heart disease, left atrial (LA) size, volume, function, and structure have clinical importance in the management of the patient [1]. In the absence of mitral valve disease and atrial myopathy, LA size commonly reflects the increased wall tension as a result of chronic volume and pressure overload [3]. Enlarged LA size is also associated with increased incidence of atrial fibrillation and stroke, risk for overall mortality after myocardial infarction, and major cardiac events or death in diabetic patients [3, 4]. Moreover, it has a comparable predictability for heart failure hospitalization and mortality as left ventricular ejection fraction [2].

LA structure is complex and it is characterized by a pulmonary venous component, a blind-ending pouch-like appendage, a vestibule which surrounds the mitral orifice, and interatrial septum [5, 6]. LA is naturally in an asymmetrical shape and its enlargement does not occur in a uniform fashion [7]. LA enlargement is constrained by the sternum and aortic root (AoR) anteriorly, tracheal bifurcation, and spine posteriorly [7–9]. Among them, AoR has more potential to change structurally and influence the structure of the LA. Dilatation is commonly seen as a pathological change of the AoR. Besides diseases, several non-hemodynamic factors are associated with AoR diameter, including age, gender, and anthropometric variables, such as height and weight and their derivatives, body surface area, and body mass index (https://www.sciencedirect.com/topics/medicine-and-dentistry/aortic-root) [10].

Echocardiography is the most commonly used imaging modality for screening and following patients with diseases involving LA morphology and function [6, 11]. Due to the fact that LA enlargement does not occur in a uniform fashion, echocardiography guidelines recommend biplane volumetric measurement of LA size [3, 7]. However, owing to simplicity and reproducibility, posterior-anterior linear measurement of LA (LAD P-A) has been used extensively in clinical practice and research [3]. The echocardiography guidelines provide methods of measurement and pitfalls regarding LAD P-A. However, there is no study that shows interference of AoR on LA structure and consequently on LAD P-A. We hypothesized that LAD P-A by echocardiography underestimates LA size in patients with AoR dilatation.

## Methods

A total of 70 subjects were prospectively enrolled in this observational study. Patients who applied to outpatient clinic for periodic follow-up or heart-related complaints were consecutively included in the study. Following medical history taking, a physical examination was performed on all subjects. Blood pressure was measured using standard aneroid sphygmomanometry in outpatient clinic. Individuals between 30 and 70 years old underwent a comprehensive echocardiographic evaluation. Patients having arrhythmia, severe valvular heart disease, chronic pulmonary disease, chest deformity, previous open chest surgery, dilated right ventricular, or left ventricular (LV) systolic dysfunction with ejection fraction (EF) LVEF < 55% were excluded. None of the patients had profoundly distorted left atrial and aortic anatomy, including in the border of these structures. The study protocol was approved by the institution’s ethical committee. The study was conducted in accordance with the Declaration of Helsinki. Echocardiogram was performed in the left-lateral decubitus position using commercially available equipment (Vivid S6 Echocardiography machine equipped with M4S-RS Probe, General Electric). Two investigators performed echocardiogram and the data for all patients were collected prospectively. More than 90% of the data was provided by one operator, the remaining by a second operator.

Aortic root (sinus of Valsalva) measurement was made in the parasternal long axis (PSLAX) view that depicts the maximum aortic diameter perpendicular to the long axis of the aorta at end-diastole using leading edge to leading edge convention [3, 12]. The same measurement protocol applied to tubular ascending aorta, where measurement point was varied between 1 and 5 cm above sinotubular junction. In case of inadequate tubular aortic view from a standard parasternal window, the transducer was moved toward the sternum till the desired image was obtained. The patients with AoR dilatation were determined on the basis of the chart displaying sex-, age-, and body size-specific upper and lower partition values for echocardiographic AoR dimensions, which was reported by the Framingham Heart Study [13].

LAD P-A was measured in the parasternal long axis view at the level of the aortic sinuses by using the leading edge to leading edge convention. Additionally, LA width (left atrial diameter from biplane apical view, minor axis, LAD) and length (long axis) were acquired both in the apical two- and four-chamber view. LA length (LAL) was determined as the shortest distance from the middle of the plane of the mitral annulus to the superior aspect of the LA [14]. LA width was determined as the distance between the lateral LA wall and interatrial septum, at the mid-atrial level [3]. LA endocardial border was traced where LA appendage and pulmonary veins were excluded in the apical two- and four-chamber view and Simpson’s method of disc was used for LA volume (LAV). All measurements relevant to the LA were made at the end of LV systole just before opening of the mitral valve [3].

M-mode echocardiography was utilized for assessment of LV end diastolic diameter, septal thickness, and EF. M-mode tracing was obtained from parasternal long axis view at the level of mitral leaflet tips [3]. LV diastolic function was assessed by using pulsed Doppler imaging of mitral flow profile, including peak early (E) and late (A) diastolic velocities in combination with septal annular velocity (e’) using Tissue Doppler imaging consistent with recommended guidelines [15]. Mitral regurgitation was graded as mild (1+), moderate (2+), moderate-severe (3+), or severe (4+) based on quantitative parameters including effective regurgitant orifice area, regurgitant volume, and vena contracta derived from color Doppler imaging. Likewise, the same quantitative parameters with addition of pressure half-time were used for assessment of aortic valve regurgitation [16, 17].

### Statistical analysis

The analyses were performed using R version 4.0.2 for statistical computing and graphics. Raw data were imported into R to determine the correlation among echocardiographic parameters, which is assessed by the Pearson correlation coefficient r. The 3D graphs represent a linear fit among 3 variables LAV, LAD, and AoR. The R code for which is lm(z ~ x+y). We run a linear model fitting for the following two models: (1) LAV_A2C_A4C_2 = b*AortRoot + c*LADP_A + err and (2) LAV_A2C_A4C_2 = b*AortRoot + c*LAD_A2C_A4C_2 + err. The color code represents the increase of the Y value. Simple linear regression analysis was performed in GraphPad Prism (ver. 8.4.3) to analyze the data in Fig. 4.

## Results

### Medical records

The demographic and echocardiographic data for those enrolled in this study are shown in Table 1. According to reference values provided by echocardiography guideline, 36 patients with LA dilatation took part in the study (https://asecho.org/wp-content/uploads/2018/08/WFTF-Chamber-Quantification-Summary-Doc-Final-July-18.pdf). Echocardiographic reference values for AoR size were reported by the Framingham Heart Study [13]. According to the chart displaying sex-, age-, and body size-specific upper and lower partition values for echocardiographic AoR dimensions, the present study includes 33 patients with AoR dilatation. Patients covered a broad range in age, weight, and height, with evidence of elevated blood pressure in some individuals. A breakdown of the study population according to cardiovascular disease condition is shown in Table 2. Most individuals exhibited hypertension, with evidence of artery disease in a few. Hyperlipidemia and artery disease were not particularly prevalent. Nearly half of the participants admitted to a history of smoking (current or past). As seen in Table 3, more than half the individuals exhibited diastolic dysfunction upon echocardiographic examination with 2 and 1 individuals having mitral and aortic regurgitation, respectively.

**Table 1:** Demographic and echocardiographic data with range, mean, median, and standard deviation

	Range	Mean	Median	SD
Age (years)	30–80	51.6	51	12.69
Weight (kg)	52–114	84.5	86	13.11
Height (cm)	150–193	172.9	172	9.11
BSA (m^2^)	1.49–2.40	1.98	1.99	0.18
Systolic BP (mmHg)	110–180	133.8	131	15.9
EF%	57–72	64.4	64	3.29
AoR diameter (cm)	2.7–4.8	3.67	3.75	0.40
LAD (cm) [cm/m^2^]	3.45–5.1 [1.65–2.94]	4.3 [2.21]	4.3 [2.18]	0.32 [0.26]
LAD P-A (cm) [cm/m^2^]	2.8–4.1 [1.28–2.27]	3.2 [1.65]	3.2 [1.63]	0.28 [0.21]
LAV (ml) [ml/m^2^]	30–111 [15.27–55.46]	69.7 [35.3]	70 [34.6]	15.11 [7.73]
LVEDD (cm) [cm/m^2^]	4.2–5.5 [2.02–2.90]	4.85 [2.46]	4.8 [2.47]	0.32 [0.21]
Septum (cm) [cm/m^2^]	0.8–1.3 [0.41–0.80]	1.09 [0.56]	1.1 [0.55]	0.10 [0.07]

*BSA*, body surface area; *BP*, blood pressure; *AoR*, aortic root; *LAD*, biplane average of left atria diameter; *LAD P-A*, posterior-anterior linear measurement of LA; *LAV*, left atrial volume; *LVEDD*, left ventricular end diastolic diameter; *EF*, ejection fraction; *SD*, standard deviation

**Table 2:** The study population with their demographic characteristics

	Number (%)
Male	62 (88.6)
Chronic disease free	21 (30.0)
Hypertension	48 (68.6)
Hyperlipidemia	8
Coronary artery disease	5
Peripheral artery disease	1
Diabetes mellitus	5
Currently smoker	25 (35.7)
Past smoker	8 (11.4)

**Table 3:** Number of disease-free individuals and patients with diastolic dysfunction, mitral, and aortic regurgitation

	No pathology	Grade 1	Grade 2	Grade 3	Grade 4
Mitral regurgitation	68	2	0	0	0
Aortic regurgitation	69	1	0	0	0
Diastolic dysfunction	25	44	1	0	0

The severity of mitral and aortic regurgitation are graded on a scale of 1 to 4, such as 1 + < 2 + < 3 + < 4+

### LA assessment

**Fig. 1:**
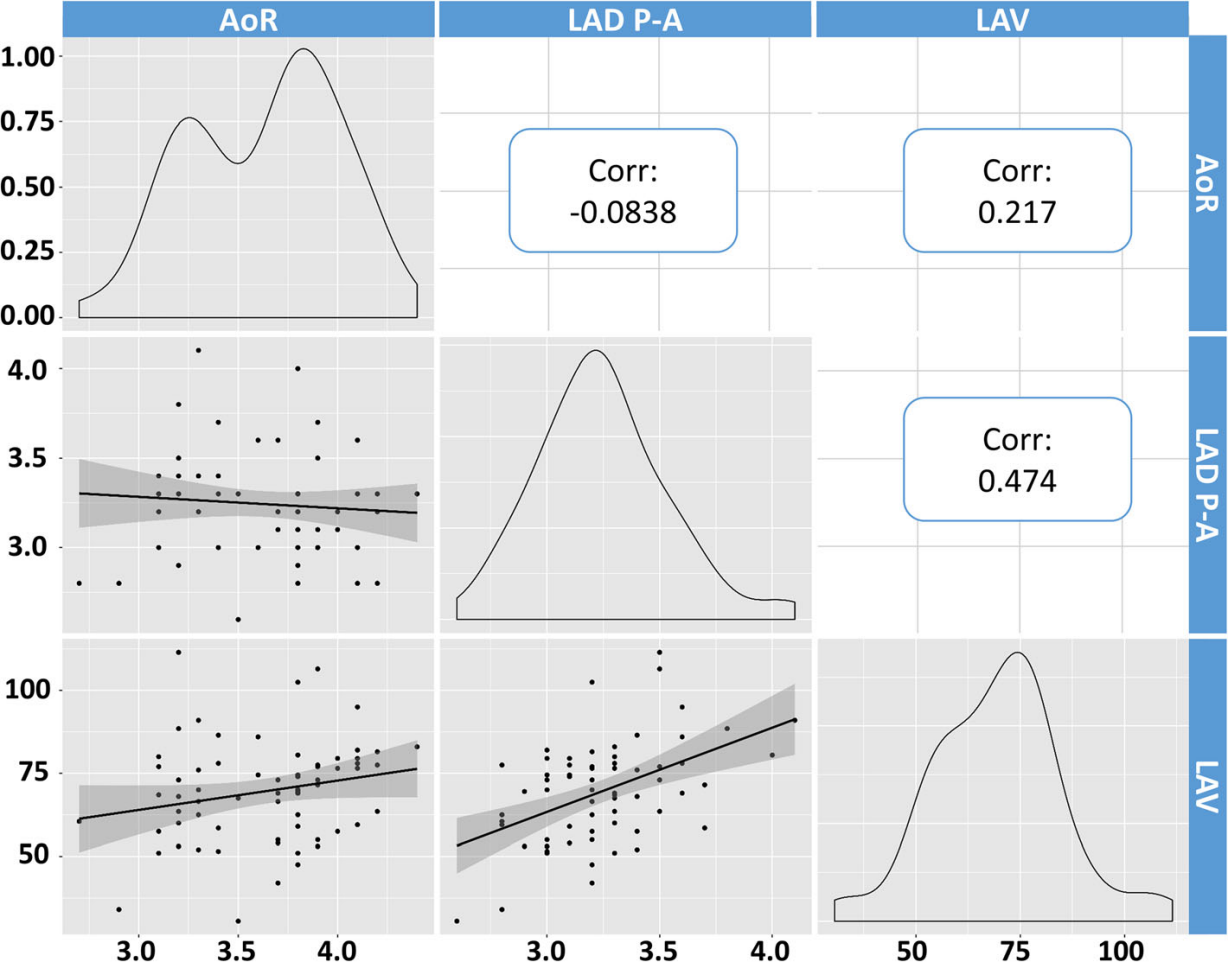
AoR, LAD P-A, and LAV data were tested for distribution and correlation. Data distribution is shown in the oblique quadrants. AoR appears to have a bimodal distribution with a pit at around 3.5 cm. LAD P-A has a unimodal distribution with a peak around 3.5 cm. LAV has a slight bimodal distribution with a pit around 65 ml (left shoulder of the highest peak). AoR showed poor correlation with LAD P-A and LAV. LAV correlation with LAD P-A results in *r* = 0.474 demonstrating a scarce degree of the two elements to vary together. The slope of the fitted lines and their 95% CI (gray/shade zone) are also represented

As seen in Fig. 1, AoR had a bimodal distribution with a pit value around 3.5 cm and poor correlation with either LAD P-A or LAV (Fig. 1). Moreover, LAD P-A had a unimodal distribution that peaked around 3.5 cm, while LAV had a slight bimodal distribution with a left shoulder, resulting in a low correlation between the two (*r* = 0.474).

**Fig. 2:**
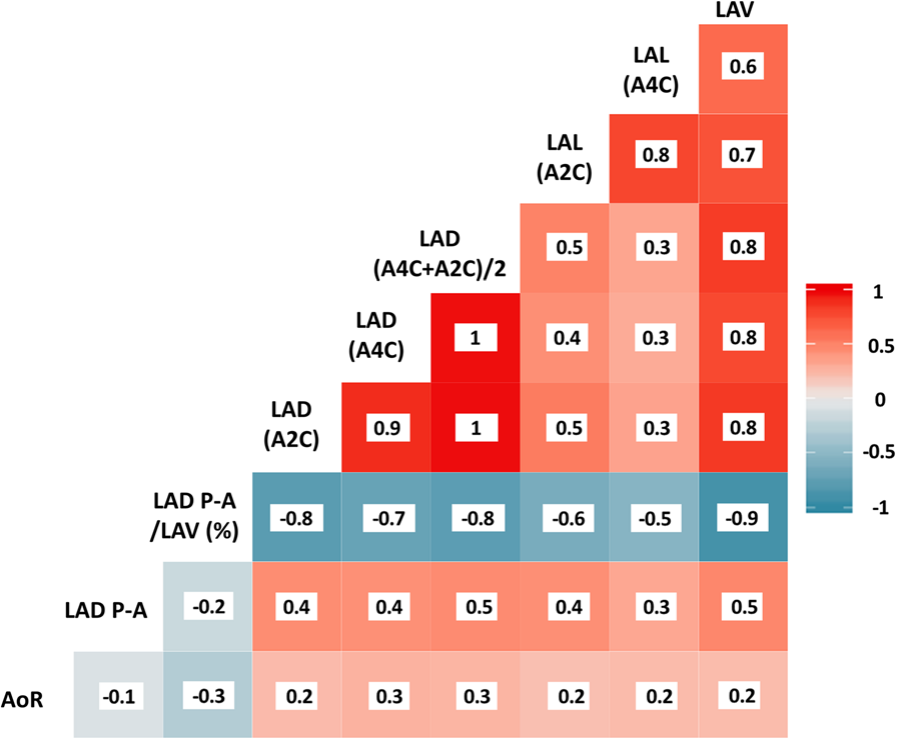
All of the echocardiographic parameters used to assess the LA size were plotted against AoR. Shown are the Pearson correlation coefficients (r values). The color code was generated to evaluate the degree of correlation (white to red = 0 to 1; white to blue = 0 to −1). LAL (A4C) and LAL (A2C) indicate the LA length from apical 4 and apical 2 chambers, respectively; LAD (A4C +A2C)/2 indicates the average of LA diameter assessed in apical 4 and apical 2 chambers; LAD (A4C) and LAD (A2C) indicate the LA diameter assessed in apical 4 and apical 2 chambers, respectively; LAD P-A/LAV (%) indicates the ratio of LAD P-A to LAV

**Fig. 3:**
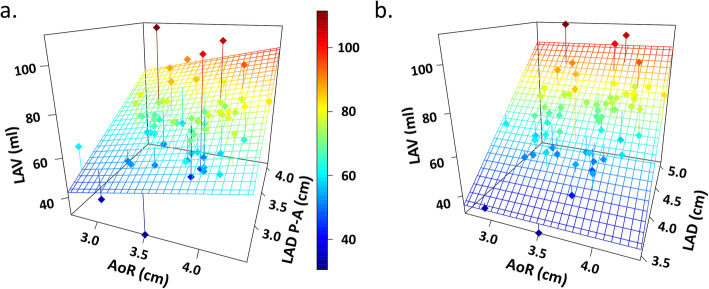
3D plots comparing the impact of increasing AoR on LAV calculated using LAD P-A or LAD. **A** The 3D graph shows that LAD P-A is diminished when AoR diameter increases. **B** The 3D graph shows that the wide range of AoR has no influence on the linear correlation between LAV and LAD. The color code is linked to the “severity” of the atrial enlargement, then red indicates those cases that are “at risk”. In graph **A**, there are less “severe” cases, but this is obviously an artifact, or in other words a technical error

The higher correlation between LAV, the touchstone for LA assessment, and other echocardiographic parameters used for LA assessment was observed when LAV was plotted against both LA diameters and volumes acquired in apical 2 and 4 chambers (Fig. 2). This is seen by the r values in the top 3 boxes of the rightmost column of Fig. 2. In contrast, LAV did not correlate well with AoR or LAD P-A (lower boxes in the rightmost column). This point is further illustrated in Fig. 3, which uses a 3D plot to compare the impact of increasing AoR on LAV calculated using either LAD P-A or LAD. The calculated LAV is shown as a colored grid. Notably, LAD P-A is diminished in range compared to LAD, likely reflecting the restricting effect of increasing AoR. Thus, the planer relationship between LAV and LAD P-A is distorted such that with increasing AoR diameter smaller volumes are calculated (Fig. 3A). LA size is underestimated and the measures that derive from it are erroneous. The 3D graph in Fig. 3B shows that the wide range of AoR has no influence on the linear correlation between LAV and LAD.

**Fig. 4:**
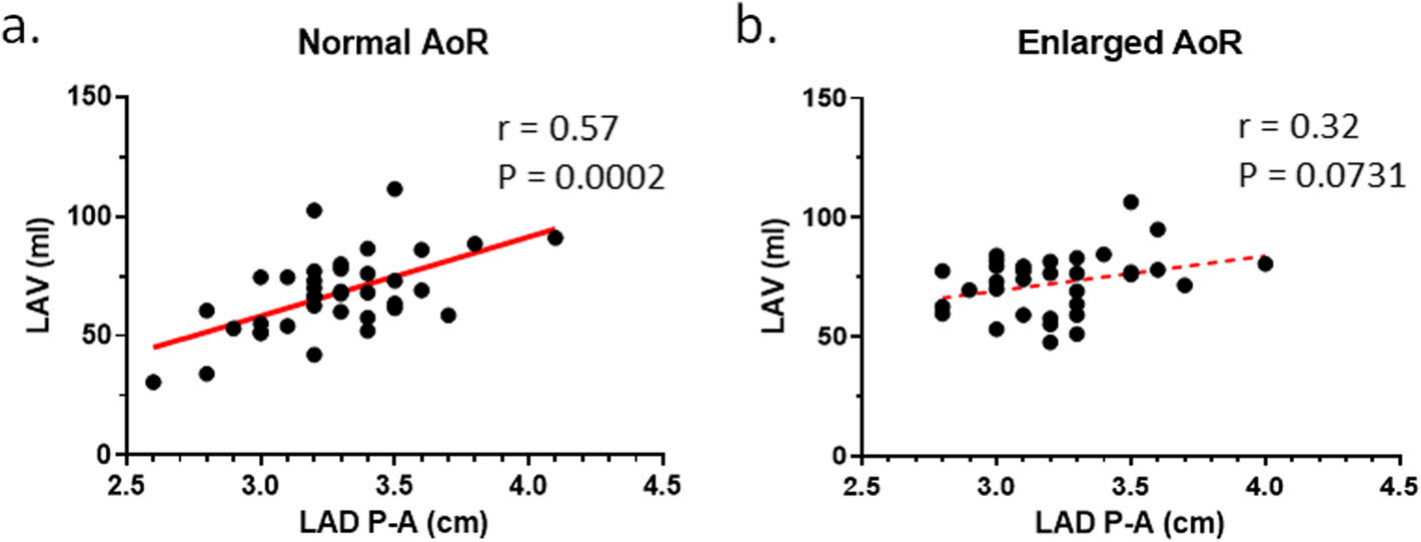
Linear regression analysis between LAD P-A and LAV. Analysis was performed for **A** normal size AoR and **B** enlarged AoR. The slope of the fitted line was significantly different from zero only for individuals with normal size aortic root

In Fig. 4, LAV was plotted as a function of LAD P-A. For those with a normal size AoR, a significant positive correlation was observed (*r* = 0.57, *P* = 0.0002) (Fig. 4A). However, for those with an enlarged AoR based on a chart including sex, age, and body surface area, no correlation was seen (*r* = 0.32, *P* = 0.0731) (Fig. 4B).

## Discussion

The present study for the first time shows that AoR diameter influences the LAD P-A. The degree of influence is enhanced as AoR diameter increases. LAD P-A is underestimated in patients with larger AoR than in those with normal AoR. AoR diameter is strongly dependent on age and body surface area [12, 13, 18]. Also blood pressure and smoking status are related to AoR dilatation [19]. Some indices of hypertension-induced target organ damage, such as LV hypertrophy, diastolic dysfunction, and carotid intima–media thickening, were found associated with echocardiographically detected AoR enlargement [10]. AoR diameter is progressively larger with aging [12], and more precisely it was reported that AoR diameter increases by approximately 1.0 mm per decade [18]. In parallel with this alteration in AoR, LA diameter also increases with age [20, 21]. LA enlargement as a burden of cardiovascular disease, which increases in prevalence with age, is common in the aged population [22]. Since LA size is an important predictor of cardiovascular disease, with the interference of aortic root, accuracy of LA becomes a more serious issue.

LA enlargement does not occur in a uniform fashion due to its naturally asymmetrical structure and/or its interaction with other anatomic structures in the thorax [7]. LA enlargement is constrained by the sternum and AoR anteriorly, tracheal bifurcation, and spine posteriorly [7–9]. Hence, LA expands predominantly in the superior-inferior (rostro-caudal) and medial-lateral (transversal) dimensions [8, 9]. LAV is derived from left atrial length and width; therefore, a restriction of LA enlargement from one side by AoR can be compensated for by enhancement of another side. As a result, LAV from apical view remains a reliable measurement in case of AoR dilatation.

In this present study, the interaction between LA size and AoR were assessed in many aspects. It was observed that AoR diameter has a significant impact on LAD-PA. However, biplane LAV was not significantly influenced by AoR diameter. This observation was graphically interpreted in Fig. 3. The 3D graph on the right shows that the wide range of AoR has no influence on the linear correlation between LAV and LAD. In contrast, the 3D graph on the left shows that LAD P-A decreases when the AoR diameter increases. Consequently, LA size is underestimated and the measures that derive from it are erroneous. The planer relationship between LAV and LAD P-A in PSLAX is distorted such that with increasing AoR diameter smaller volumes are calculated.

The current study was designed to determine the correlation and accuracy of apical 2 and 4 chambers compared to LAD P-A. A larger sample size will potentially allow for the cut-off point for the impact of increased AoR for determination of LA size from LAD P-A to be established. The impact of enlarged AoR might not be limited to structural alteration of LA, and function and action potential propagation of LA might also be affected from the interaction between AoR and LA wall. Further studies using cardiac magnetic resonance imaging and 3-dimentional echocardiography with spackle tracking can provide a detailed picture of the LA and possible functional alterations.

## Conclusion

Firstly, the data from this study indicates that LAD P-A underestimates the LA size in patients with AoR dilatation. Based on the findings of this study, we highly recommend not determining LA size in PSLAX in patients with AoR dilatation. Secondly, the present study offered an insight into the relationship between AoR and LA, which can constitute a basis for further investigations using more advanced imaging modalities.

## Data Availability

The datasets used and/or analyzed during the current study are available from the corresponding author on reasonable request.

## Abbreviations

AoR: Aortic root
EF: Ejection fraction
LA: Left atrial
LAD P-A: Posterior-anterior linear measurement of LA
LAD: Left atrial diameter from biplane apical view, minor axis
LAV: LA volume
LAL: LA length
LV: Left ventricular
PSLAX: Parasternal long axis
